# An introduction to the WHO 5th edition 2022 classification of testicular tumours

**DOI:** 10.1111/his.14675

**Published:** 2022-07-01

**Authors:** Daniel M Berney, Ian Cree, Vishal Rao, Holger Moch, John R Srigley, Toyonori Tsuzuki, Mahul B Amin, Eva M Comperat, Arndt Hartmann, Santosh Menon, George J Netto, Mark A Rubin, Samra Turajlic, Maria R Raspollini, Satish K Tickoo

**Affiliations:** ^1^ Department of Molecular Oncology Barts Cancer Institute, Barts and The London School of Medicine and Dentistry, QMUL and Barts Health NHS Trust London UK; ^2^ WHO Classification of Tumours Group International Agency for Research on Cancer, World Health Organization Lyon France; ^3^ Basavatarakam Indo American Cancer Hospital and Research Institute Hyderabad India; ^4^ Department of Pathology and Molecular Pathology University Hospital Zurich and University Zurich Zurich Switzerland; ^5^ McMaster University Hamilton ON Canada; ^6^ Department of Surgical Pathology Aichi Medical University, School of Medicine Nagakute Japan; ^7^ Department of Pathology and Laboratory Medicine The University of Tennessee Health Sciences Center Memphis TN USA; ^8^ Department of Urology University of Southern California, Keck School of Medicine Los Angeles CA USA; ^9^ Department of Pathology General Hospital, Medical University Vienna Austria; ^10^ Department of Pathology Tenon Hospital Sorbonne University Paris France; ^11^ Institute of Pathology University Hospital Erlangen, Friedrich‐Alexander University Erlangen Germany; ^12^ Tata Memorial Centre Homi Bhabha National Institute Mumbai India; ^13^ University of Alabama at Birmingham, Pathology Birmingham AL USA; ^14^ University of Bern, Director of the Department for BioMedical Research Bern Switzerland; ^15^ The Francis Crick Institute London UK; ^16^ Histopathology and Molecular Diagnostics University Hospital Careggi Florence Italy; ^17^ Memorial Sloan‐Kettering Cancer Center, Pathology New York NY USA

**Keywords:** classification, Germ Cell Tumour, teratoma, testis, WHO

## Abstract

The 5th edition of the World Health Organisation Blue Book was published recently and includes a comprehensive update on testicular tumours. This builds upon the work of the 4th edition, retaining its structure and main nomenclature, including the use of the term ‘germ cell neoplasia in situ’ (GCNIS) for the pre‐invasive lesion of most germ cell tumours and division from those not derived from GCNIS. While there have been important developments in understanding the molecular underpinnings of testicular cancer, this updated classification paradigm and approach remains rooted in morphology. Nomenclature changes include replacement of the term ‘primitive neuroectodermal tumour’ by ‘embryonic neuroectodermal tumour’ based on the non‐specificity of the former term and to separate these tumours clearly from Ewing sarcoma. Seminoma is placed in a germinoma family of tumours emphasising relation to those tumours at other sites. Criteria for the diagnosis of ‘teratoma with somatic transformation’ have been modified to not include variable field size assessments. The word ‘carcinoid’ has been changed to ‘neuroendocrine tumour’, with most examples in the testis now classified as ‘prepubertal type testicular neuroendocrine tumour’. For sex cord‐stromal tumours, the use of mitotic counts per high‐power field has been changed to per mm2 for malignancy assessments, and the new entities, ‘signet ring stromal tumour’ and ‘myoid gonadal stromal tumour’, are defined. Well‐differentiated papillary mesothelial tumour has now been defined as tumour type with a favourable prognosis. Sertoliform cystadenoma has been removed as an entity from testicular adnexal tumours and placed with Sertoli cell tumours.

## Graphical abstract

The WHO 2022 classification changes include new nomenclature such as embryonic neuroectodermal tumour. There are new entities such as pre‐pubertal type testicular neuroendocrine tumour, signet ring stromal tumour and myoid gonadal stromal tumour. Evaluation of tumours by mitotic counts per mm^2^ is now mandated.

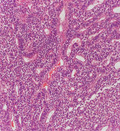

## Introduction

Testicular tumour pathology is challenging for two main reasons. First, these neoplasms are relatively rare; secondly, they are extremely diverse, meaning that the rarer entities may be encountered extremely infrequently by a diagnostic general histopathologist, or even by a specialist in genitourinary (GU) pathology. As some tumour types will be encountered less than once a year or even once a decade, it is unsurprising that few can gain experience in this area.

There have been numerous previous classifications of testicular tumours from a variety of panels and resources. For many years the British Testicular Tumour panel was widely used in many countries.^1^ The World Health Organisation (WHO) publications in the 21st century have helped to unify this field.^2^ This especially pertains to the schism which had developed between those using different nomenclatures for the pre‐neoplastic lesion of testicular germ cell tumours (previously ‘carcinoma *in situ*’, ‘intratubular germ cell neoplasia, unclassified’ and ‘testicular intra‐epithelial neoplasia’), that undoubtedly has caused confusion. The resolution of this rift and now near‐universal use of germ cell neoplasia *in‐situ* (GCNIS) as the nomenclature has united many and has healed large divisions.^3^

This 2022 WHO classification^4^ builds upon the radical revision made in 2016, especially to the germ cell tumours. It also has been adapted to the new format of the 5th edition of the classification.

This hierarchical classification has had to make some compromises along the way, as the scheme of ‘category’, ‘family’, then ‘type’ then ‘subtype’ with a possibility of different patterns does not fit neatly, especially in the diversity of germ cell tumours. As has been noted in other publications of the WHO Blue Books in the fifth series, the term ‘variants’ is reserved for variable genomic mutations.

The straightforward subdivision of germ cell tumours into the vast majority, derived from GCNIS and those unrelated to it, has been retained. The other non‐invasive components are of generally less importance and only minor changes have been made.

Added to the non‐invasive lesions derived from GCNIS is the other pre‐neoplastic lesion in the testis, gonadoblastoma. Although often defined as a mixed sex cord‐stromal tumour, it is composed of neoplastic germ cells set in a matrix of immature sex cord cells. These tumours occur in dysgenetic gonads in patients who possess at least a portion of the Y‐chromosome, but are often phenotypically female. Although this entity is well known and understood with a very high propensity for transformation into invasive seminoma, it may cause diagnostic challenges. The dissecting variant may mimic seminoma and lead to overdiagnosis and potential overtreatment.^5^, ^6^

After some debate, we have not included the recent intriguing descriptions of non‐gonadoblastoma mixed germ cell/sex cord tumours in the classification. In previous classifications these were thought to be due to collision of sex cord‐stromal tumours with scattered germ cells. A tumour with an adult granulosa cell tumour component and spermatocytic tumour has been described, one of which invaded outside the testis.^7^ This defies easy classification and the authors feel, on balance, that these are collision tumours rather than a combined tumour with neoplastic components in both cell lines.

## Conclusions

The new WHO 5th edition builds upon the work of the 4th edition, retaining its main elements while hopefully clarifying areas of outdated nomenclature and introducing a limited number of new entities. While there have been important developments in understanding the molecular underpinnings of testicular cancer, this updated classification paradigm and approach remains rooted in morphology. The authors hope that the questions and uncertainties raised here will be taken up as scholarship opportunities by the GU pathological community to aid treatment decisions in this challenging area.

## Seminoma

Although the term ‘seminoma’ remains unchanged, in this edition we wished to raise the issue of nomenclature, not just in the testis but in germ cell tumours in any organ.

The terms dysgerminoma, seminoma and germinoma as diagnoses have been used for the same tumour with the similar appearances, variations, immunochemistry and molecular pathology throughout the body. We would like to suggest that a greater unification of terminology would add to better consistency, especially for non‐pathologists who have to treat this disease.

To this end, we have placed seminoma in the ‘germinoma’ family of tumours in the classification. We hope that this will aid any future attempts to create a unified classification, independent of tumour site.^8^

### Subtypes and patterns of seminoma

**Figure 1:**
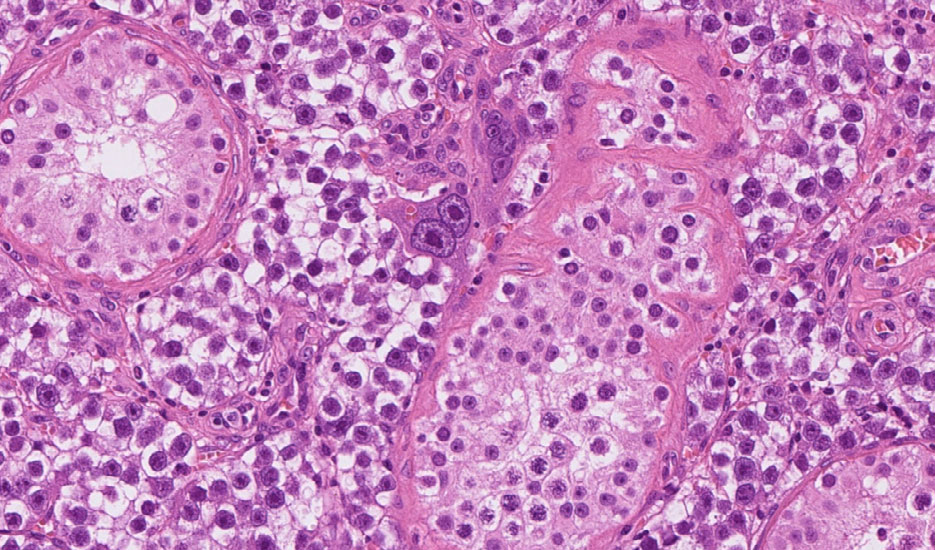
Seminoma with a syncytiotrophoblastic cell. Seminomas are now placed in the germinoma family. [Colour figure can be viewed at wileyonlinelibrary.com]

Although seminoma is the sole type of tumour in the germinoma category in the testis it may show many patterns, with more or less inflammation, granulomata, tubular morphology or even signet‐ring features. However, we have used only one subtype of seminoma: seminoma with syncytiotrophoblastic cells (Figure 1). These may present with mildly raised serum beta‐ human chorionic gonadotrophin, and awareness of this occurrence is helpful. This has been implemented for practical reasons, as it is still occasionally misdiagnosed as choriocarcinoma, and cases are prone to be inappropriately treated.^9^ This subtype behaves in an identical manner to seminoma with equally high chemo‐ and radio‐sensitivity, and treatment on a non‐seminomatous type protocol can be avoided.

We would add that subclassification of seminoma on the basis of better or worse prognosis of the various patterns would be extremely unlikely. The exceptionally good prognosis of seminoma means that powering any future study statistically is immensely difficult without thousands or even tens of thousands of cases.

## Non‐seminomatous germ cell tumours

Changes in non‐germinomatous tumours are again minimised. Due to the nature of the classification, it is not possible to have a ‘subfamily’ of trophoblastic tumours within the non‐germinomatous category, although this would have been the preferred option. Embryonal carcinoma, yolk sac tumour and the trophoblastic tumours are essentially unchanged in this classification. However, we have instituted a number of changes in the teratoma with somatic transformation category.

First, an important principle of the 5th edition is to use mm^2^ rather than number of high‐ or low‐power fields to make comparisons in mitotic rates between different microscopes comparable, and to avoid artificial variations.^10^ A persistent problem in testicular tumour classification has been because while the number of fields has been given, no diameters of the fields has been provided in the cited papers. We would encourage future publications to make all measurements in mm.^2^ While the diagnosis was previously established by using the definition ‘a nodule of malignant cells equivalent to area seen under 4× objective or expansile nodule overgrowing other GCT elements,’ in the 5th edition the size criterion has been changed to the area occupied by a 5‐mm diameter field.

**Figure 2:**
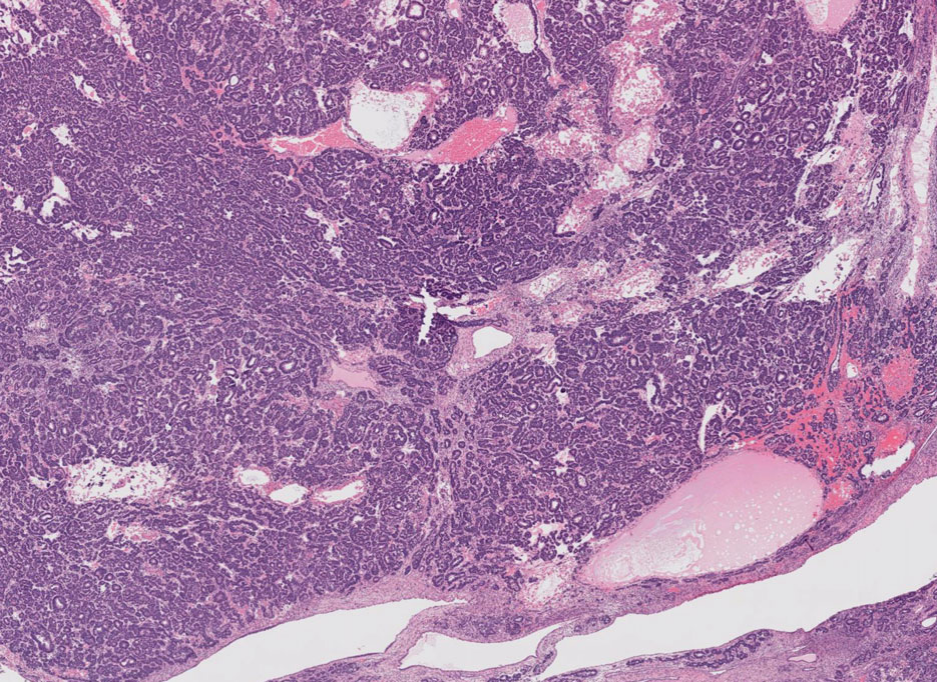
Testis showing a teratoma with somatic type malignant transformation to nephroblastoma. [Colour figure can be viewed at wileyonlinelibrary.com]

This comes with several caveats. It is likely that this may more effectively apply to metastases rather than ‘primary’ somatic transformations within the testis. It is possible that this diagnosis will be refined in future iterations. Secondly, this 5‐mm size is an absolute minimum and it should be pure, without any admixed germ cell component. The nephroblastomatous somatic transformation illustrated is a rare example of this process. (Figure 2).

**Figure 3:**
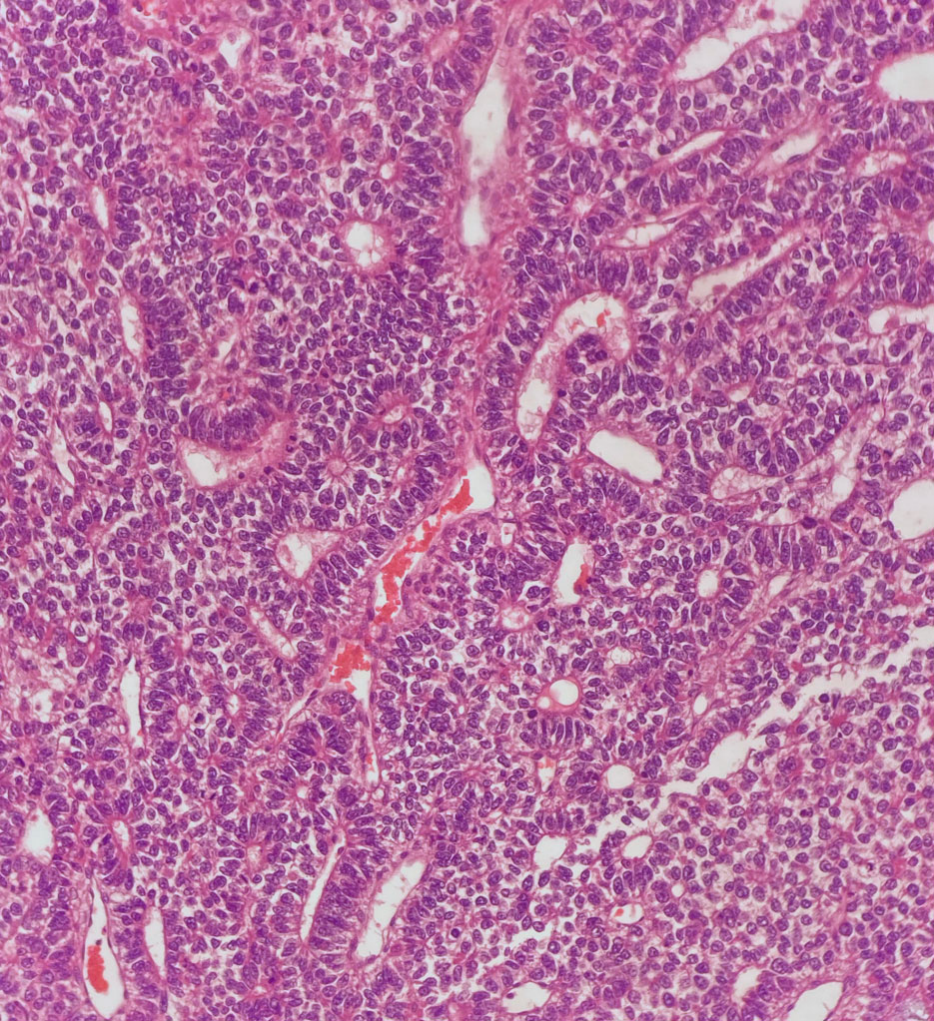
Embryonic neuroectodermal tumour. [Colour figure can be viewed at wileyonlinelibrary.com]

In testicular post‐pubertal teratomas (and also in so called immature teratomas of the ovary) there are frequently areas of small round blue cells with neural differentiation, which may somatically transform to a dominant neoplastic proliferation (Figure 3). It has been known for many years that after metastasis these may be intractable to therapy and have a poor prognosis, although they have a varied morphology with Homer–Wright rosettes, tubules or appear to be more solid in appearance.^11^, ^12^

The nomenclature ‘primitive neuroectodermal tumour’ (PNET) was originally applied to this diverse group at many anatomical sites. However, that is now known to represent a wide range of neoplasms with differing prognoses, immunochemistry and molecular changes. As identified in germ cell tumours of the testis, the nomenclature of PNET has now become out of line with the classification of neuroectodermal elements in the brain and other sites.

In 2016 the move in the central nervous system (CNS) WHO classification to a more molecularly aligned classification saw the term PNET replaced by ‘embryonal tumour with multilayered rosettes, C19mc altered or NOS,^13^ and this has been shortened to ‘embryonal tumour with multilayered rosettes’ in the latest classification in 2021.^14^ PNET was also used previously as a synonym for Ewing sarcoma, but this was also removed in 2013 in bone and soft tissue tumours.^15^, ^16^

The PNET‐like elements and pure PNET tumours seen in testicular and ovarian tumours has caused frequent confusion in the GU and gynaecological literature. This can lead to misdiagnosis and mistreatment.

These embryonic foci do not show the Ewing sarcoma translocation of ESWR1::FLI1 and are more akin to their CNS counterparts.^11^, ^17^, ^18^ Given the fact that embryonal carcinoma exists as a well‐established entity in the testis, using the CNS terminology of embryonal tumour for testicular PNET could lead to confusion, particularly to non‐pathologists. Therefore, the term ‘embryonic‐type neuroectodermal tumour’ (ENET) has been used to replace PNET as an entity in the testis and in ovarian tumours.^19^ Where the somatic transformation in the form of a pure mass has not occurred, these can be mentioned as areas of ‘ENET’‐like elements within teratoma in both the primary and at metastatic sites. It is to be hoped, as previously suggested by one of the authors, that this will encourage reconsideration of the term ‘immature teratoma’ in the ovary^8^ and be replaced with the more specific ‘ovarian teratomas with ENET‐like areas’. This classification is also being adopted by the AFIP guidelines in a unified approach to testicular tumour classification.

It should be remembered that Ewing sarcoma has rarely been identified previously in the testis.^20^ These tumours lack germ cell tumour components and show strong diffuse membranous positivity for CD99 and nuclear Fli‐1.^21^ Unlike ENET, they are negative for GFAP and testing will reveal the typical fusion translocation gene. Such tumours are much rarer than ENET in the testis.

## Germ cell tumours unrelated to GCNIS

This smaller category remains but is vitally important, so that correct management is instituted.

Spermatocytic tumour nomenclature remains unchanged from the previous edition, reinforcing its generally indolent nature. Although metastases are almost always associated with sarcomatous transformation, it is notable that two recent case reports describe hybrid entities which are spermatocytic tumours capable of metastasis but which show isochromosome 12p, which is associated with the GCNIS‐related tumours.^22^, ^23^ These rarities certainly seem to be opposed to the current classification, but further investigation will be required in order to refine the current status. However, changes have been made to the classification of tumours with differentiated neuroendocrine components, and a new type has been added: ‘pre‐pubertal type testicular neuroendocrine tumour’.

In alignment with WHO guidance in most other anatomical sites,^24^ we have discarded the ‘carcinoid’ nomenclature throughout the GU book. To avoid as much repetition as possible there is a separate neuroendocrine tumour chapter. However due to its close association with teratoma, testicular neuroendocrine tumours are described in the main classification section.

**Figure 4:**
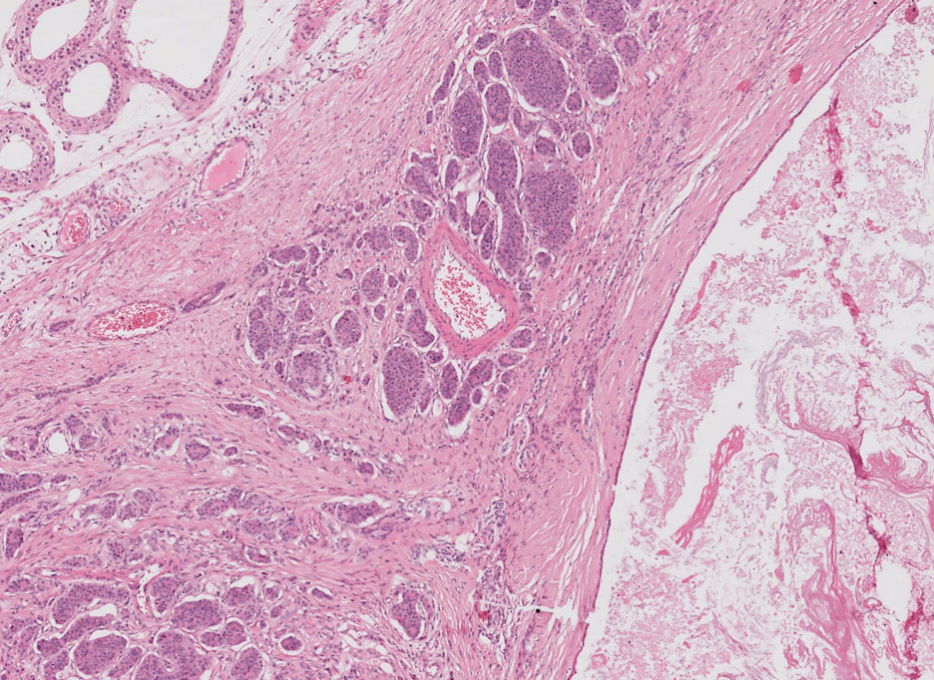
A pre‐pubertal type teratoma with and area of low‐grade neuroendocrine tumour. [Colour figure can be viewed at wileyonlinelibrary.com]

**Figure 5:**
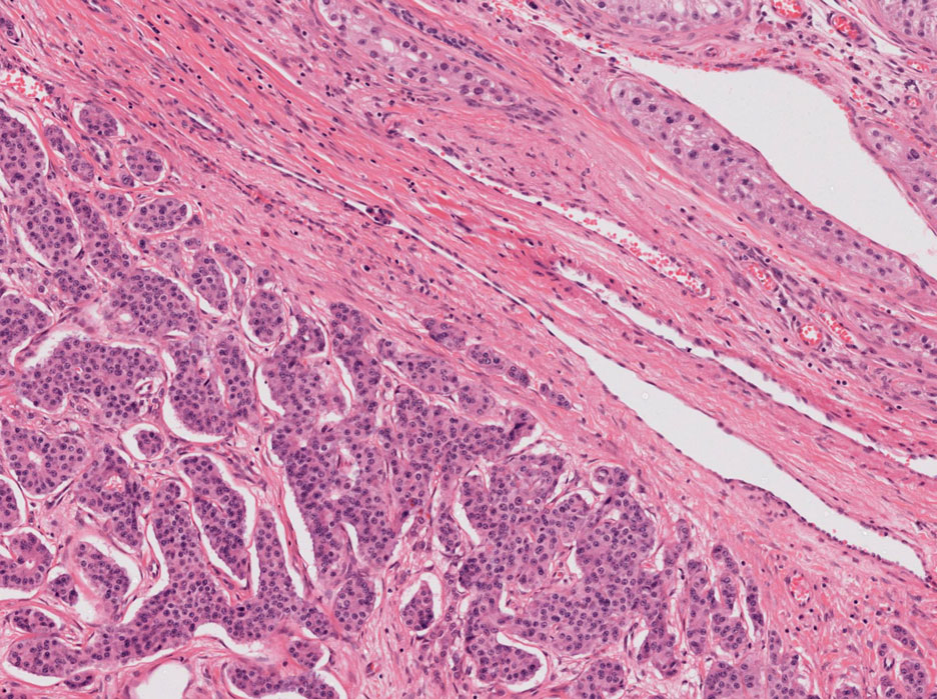
Pre‐pubertal type testicular neuroectodermal tumour. [Colour figure can be viewed at wileyonlinelibrary.com]

It has become apparent that most testicular neuroendocrine tumours (NETs) arise in the setting of a pre‐pubertal teratoma (Figures 4 and 5) and are associated with them.^25^, ^26^ Therefore, we have added this type to the classification (Table 1).

**Table 1:** Major changes to the 2022 WHO 5th edition of testicular tumours

Primitive neuroectodermal tumour renamed embryonic neuroectodermal tumour
Seminoma placed in germinoma family of tumours
Criteria for teratoma with somatic transformation changed including size‐based rather than on low‐power fields criteria
Carcinoid tumours of the testis now termed pre‐pubertal type testicular neuroendocrine tumour (with acknowledgement of rare post‐pubertal type NETS)
Use of mitotic counts per HPF changed to per mm^2^ for malignancy assessment in sex cord‐stomal tumours
Signet ring stromal tumour defined as a new entity in the WHO classification
Myoid gonadal stromal tumour has been moved from a provisional entity to a new entity
Well differentiated papillary mesothelial tumour defined as a separate tumour type
Sertoliform cystadenoma removed from adenexal tumours and placed with Sertoli cell tumours

HPF, high‐power field; WHO, World Health Organisation; PNET, primitive neuroectodermal tumour.

We acknowledge that occasional NETS arise in post‐pubertal type teratomas and are associated with GCNIS.^27^ To avoid repetition, we have included this possibility within the teratoma with somatic transformation category of GCNIS‐related tumours. There is no evidence base on size criteria for NET ‘somatic transformation’ criteria. Further work is required on the prognosis of testicular NETS and, as at other sites, mitotic index and Ki‐67 may be of importance. The vast majority have a low Ki‐67 and mitotic count and do well.^28^, ^29^ Again, we encourage use of mm^2^ to assess proliferation in future series.

## Sex cord‐stromal tumours

Sex cord‐stromal tumours have caused unique problems for their classification due to their great heterogeneity in morphology, immunohistochemistry and genetic associations. Sertoli cell tumours in particular show a huge range of variations that cause confusion and difficulties.

Also, there are challenges in terms of the assessment of malignancy which are yet to be resolved, and may influence clinical management.^30^ Although rare, the diagnosis of sex cord‐ stromal tumours may be increasing secondary to the rise in detection and later excision of ultrasound detected masses of dubious clinical significance.^31^ The malignancy rates vary secondary to local clinical practice.

There is now a separate chapter on genetic tumour syndromes, and as intratubular hyalinising Sertoli cell tumour occurs exclusively in Peutz–Jeghers syndrome, this has been described under genetic tumour syndromes.^32^ In contrast, large cell calcifying Sertoli cell tumour is associated with Carney complex, but sporadic cases occur. It is thus described in both sections.^33^

**Figure 6:**
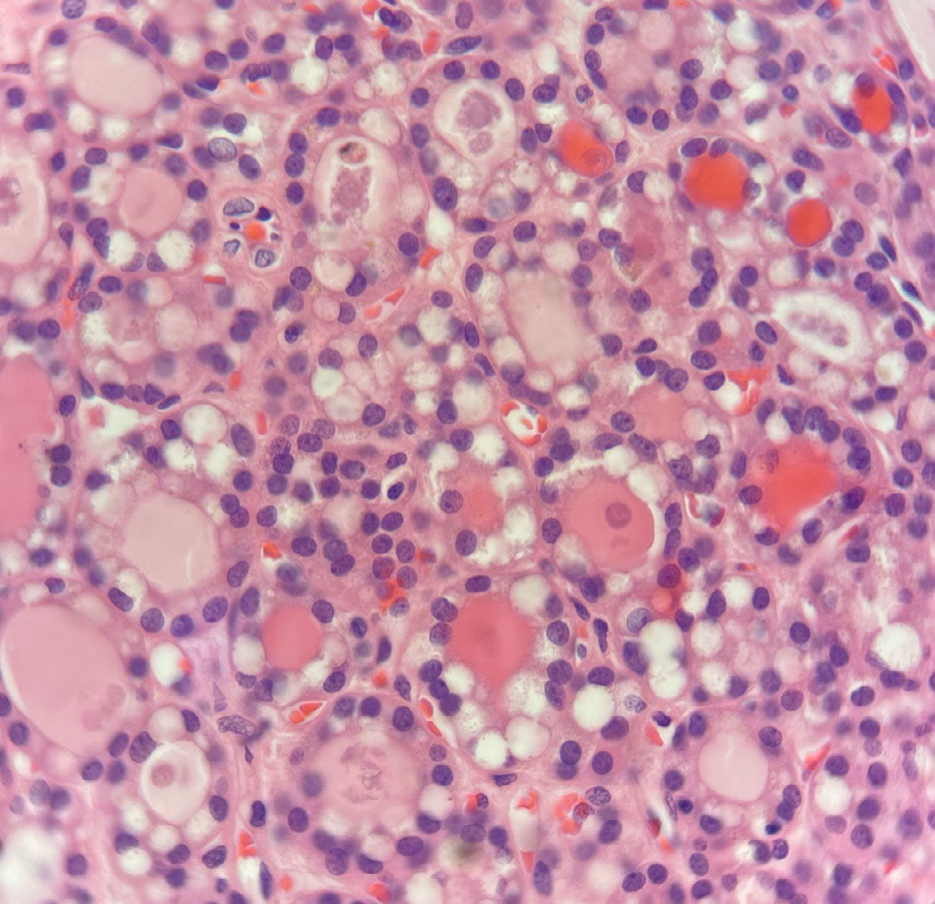
Signet ring stromal tumour. [Colour figure can be viewed at wileyonlinelibrary.com]

Occasional sex cord‐stromal tumours show a striking signet ring pattern. This may cause confusion and raise a concern of metastatic disease from the GI tract in particular.^34^ For this reason, they have been classified here as a separate entity as ‘signet ring stromal tumour’ (Figure 6). They appear to behave in an indolent fashion and are positive for beta catenin. There is some debate on whether this is just a pattern of Sertoli cell tumour,^35^, ^36^ but as it can cause clinical concern it was thought appropriate to classify them separately in order to raise awareness of this entity.

**Figure 7:**
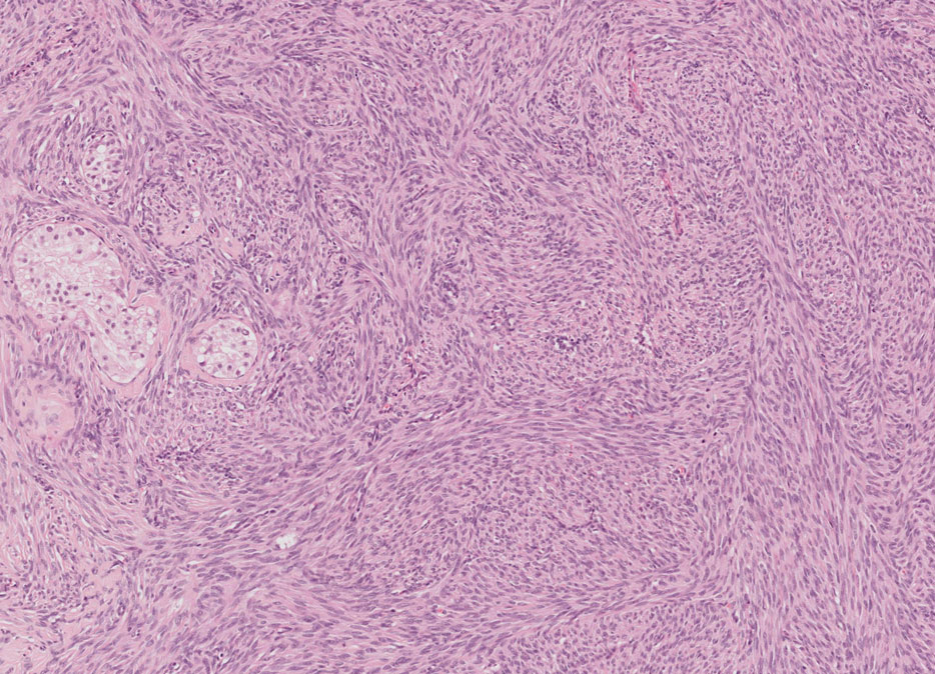
Myoid gonadal stromal tumour. [Colour figure can be viewed at wileyonlinelibrary.com]

Myoid gonadal stromal tumour was an emerging entity in the previous edition and has been instated as a full entity in this classification. It does appear to be morphologically and immunochemically distinct from other sex cord‐stromal tumours, being more related to myoid cells in association with seminiferous tubules (Figure 7). It also appears to behave in a benign fashion.^37^, ^38^, ^39^

Clarification on the occasional sex cord‐stromal tumours which led to multiple terminologies, including the term ‘unclassified SCST’. It was thought that the word ‘unclassified’ could be mistaken for ‘undifferentiated’ or imply poor differentiation. There is a separate ‘mixed SCST’ for tumours with a mixture of different elements, and SCST, NOS when the tumour appears to be SCST in origin, but specific differentiation patterns are not well seen.^40^, ^41^

The treatment and follow‐up of SCSTs remains debatable, as even though the vast majority behave in a benign fashion, malignancy may be unpredictable.^42^ Further work in this area is needed, especially as there are no effective chemotherapy or radiotherapy treatment options.

Factors which are high risk in predicting metastasis in the literature include size greater than 50 mm, necrosis, nuclear atypia, vascular invasion, invasion outside the testis and the criterion of a raised mitotic count: unfortunately, more than five mitoses per 10 high‐power fields.^30^ One recent study has attempted a scoring system to synthesise these different criteria in Leydig cell tumours.^43^

As mentioned previously, as publications have not specified microscopic field diameters, the calculation of this to mitotic counts per mm^2^ will require further work on retrospective or prospective series.

## Adnexal tumours

The complexities of the tumours of the testicular adenexae have been partially resolved by the institution of a separate section on rare mesenchymal tumours which occur throughout the GU tract. However there remains a whole constellation of entities unique to the area.

Two changes are worth highlighting. First, in the mesothelial tumours we have separated as a type the ‘well‐differentiated papillary mesothelial tumour’ (WDPMT) from adenomatoid tumour and malignant mesothelioma of the tunica vaginalis (Figure 6). It is thought that when strictly diagnosed with no invasive foci,^44^ WDPMT behaves in an indolent fashion.^45^

Secondly, the very rare Sertoliform cystadenoma, which primarily occurs in the rete testis, has also been subsumed into the Sertoli cell tumour category. These tumours show overlapping morphology and immunohistochemistry to Sertoli cell tumours and are only differentiated by their origin in the rete testis leading to their unusual morphological appearance.^44^ Until definitive immunohistochemical or molecular distinction is shown, it has been removed from testicular adnexal tumours. They may originate from cells at the junction of seminiferous tubules and rete testis that can differentiate towards sex cord‐stromal cells.

## Author contributions

All the authors were editors of the WHO 2022 testis classification and provided intellectual input into the classification.

## Data Availability

NA.
